# Potential impact of European Medicines Agency measures to minimize risk of serious side effects on JAKi prescribing and utilization in the UK

**DOI:** 10.1093/rheumatology/keae279

**Published:** 2024-05-15

**Authors:** Zixing Tian, Lianne Kearsley-Fleet, James Galloway, Kath Watson, BSRBR-RA Contributors Group, Mark Lunt, Kimme L Hyrich

**Affiliations:** Centre for Musculoskeletal Research, Division of Musculoskeletal and Dermatological Sciences, School of Biological Sciences, Faculty of Biology Medicine and Health, The University of Manchester, Manchester Academic Health Science Centre, Manchester, UK; Centre for Musculoskeletal Research, Division of Musculoskeletal and Dermatological Sciences, School of Biological Sciences, Faculty of Biology Medicine and Health, The University of Manchester, Manchester Academic Health Science Centre, Manchester, UK; Centre for Rheumatic Diseases, King’s College London, London, UK; Centre for Musculoskeletal Research, Division of Musculoskeletal and Dermatological Sciences, School of Biological Sciences, Faculty of Biology Medicine and Health, The University of Manchester, Manchester Academic Health Science Centre, Manchester, UK; Centre for Musculoskeletal Research, Division of Musculoskeletal and Dermatological Sciences, School of Biological Sciences, Faculty of Biology Medicine and Health, The University of Manchester, Manchester Academic Health Science Centre, Manchester, UK; Centre for Musculoskeletal Research, Division of Musculoskeletal and Dermatological Sciences, School of Biological Sciences, Faculty of Biology Medicine and Health, The University of Manchester, Manchester Academic Health Science Centre, Manchester, UK; National Institute of Health Research Manchester Biomedical Research Centre, Manchester University NHS Foundation Trust, Manchester, UK

**Keywords:** JAK inhibitor, rheumatoid arthritis, real-world evidence

## Abstract

**Objective:**

Janus kinase inhibitors (JAKis) or targeted synthetic (ts) disease-modifying antirheumatic drugs (DMARDs) effectively treat rheumatoid arthritis (RA). However, due to safety concerns, the European Medicines Agency (EMA) published risk-minimization measures limiting JAKi prescription to certain at-risk patients unless no suitable alternative is available. This analysis included patients who had started their first-ever JAKi (before EMA measures were published) in a large national cohort study to investigate the potential impact of these measures on JAKi prescribing and utilization in the UK.

**Method:**

RA patients starting first-ever JAKi therapy in BSRBR-RA between 13 February 2017 and 31 May 2022 were included. The percentages of patients meeting the EMA risk criteria were presented. For the at-risk patients, their previous numbers of distinct biologic (b) DMARD classes prescribed were described.

**Result:**

A total of 1341 patients were included, and 80% (*N* = 1075) met ≥1 EMA risk criterion. Of those who met ≥1 risk criterion, 529 patients (49%) had received JAKi as their first or second b/tsDMARD class, whereas 299 (28%) had received ≥3 prior bDMARD classes.

**Conclusion:**

Four-fifths of RA patients who had commenced a JAKi before the EMA advisory were considered ‘at-risk’, with prescribing only advised if there was no suitable alternative. Almost a third of those patients had already received ≥3 bDMARDs classes, and alternative therapies would be very limited for them; however, suitable alternatives might have existed for the remaining proportion, especially for those who received a JAKi as their first or second b/tsDMARD, and re-evaluation of the suitability of their treatment may be needed.

Rheumatology key messages

- Four-fifths of RA patients commencing a JAKi before the EMA advisory were considered ‘at-risk’, and prescribing was advised only if no suitable alternative was available.
- Almost a third of those patients had already received ≥3 bDMARDs classes, leaving limited alternative options.
- For the remaining patients, there may have been suitable alternatives available, warranting a re-evaluation of their treatment suitability.

## Introduction

Four Janus kinase inhibitors (JAKis), baricitinib, tofacitinib, upadacitinib and filgotinib, have been approved to treat RA as a further therapeutic option in the UK and are categorized as targeted synthetic DMARDs (tsDMARDs) [1]. When the first JAKi for treating RA, tofacitinib, was approved at a dose of 5 mg twice daily by the U.S. Food and Drug Administration in 2012, an additional post-marketing clinical trial was mandated because of a potential increased risk of cancer, cardiovascular diseases, and serious infections [2, 3]. Thus, the ORAL Surveillance trial was designed to evaluate the risk of cardiovascular disease and cancers associated with tofacitinib treatment in RA patients with pre-existing increased cardiovascular risk. The results indicated a safety signal for increased risk of major adverse cardiovascular events (MACEs) and cancer in tofacitinib *vs* TNF inhibitors (TNFis) [4]. Post-hoc analyses of ORAL Surveillance found that, compared with TNFis, tofacitinib showed: (1) a signal of higher MACEs in patients with a history of atherosclerotic cardiovascular disease, although not statistically significantly higher; and (2) a signal of higher MACEs and cancer rates in patients older than 65 years who had a long smoking history [5, 6]. In addition, in a 14 multi-database real-world study in RA patients, baricitinib was associated with a significantly increased risk of venous thromboembolism, and a numerically greater risk of a MACE and serious infections, compared with TNFis [7], raising concerns about all JAKi therapies.

These safety concerns prompted the European Medicines Agency (EMA) to publish new measures to minimize the risk of serious side effects. They advise against using JAKis in certain patient subgroups unless no suitable alternative is available [8], including patients aged 65 years or above, and those patients with certain high-risk features, including cardiovascular or malignancy risk or long smoking history. In the UK, there are four biologic (b)DMARD classes: TNFis, B cell-depleting agents (anti-B cell agents), IL-6 inhibitors (IL-6is), and a T cell co-stimulation blocker (CTLA4) [9], also recommended for advanced RA treatment. All may be used as a first-line b/tsDMARD following ineffectiveness or contradictions/adverse events with at least two conventional synthetic (cs)DMARDs, with the exception of rituximab (anti B cell), which is licensed for use following failure of previous b/tsDMARD(s). However, a recent analysis of UK registry data has shown that TNFis remain the most common first-line b/tsDMARD [10], likely related to both physician experience and cost, whereas a JAKi is often used later in patients who have already tried other bDMARDs, in part related to the fact that JAKis are the most recently introduced advanced therapies for RA, and, therefore, many patients have already received other bDMARDs. It is not currently known what impact the EMA minimization measures may have on JAKi prescribing and what proportion of patients receiving JAKis could be classified as higher-risk patients, and thus, treatment would be relatively contraindicated and therefore, potentially stopped.

This analysis aimed to describe, among a cohort of patients who had already started a JAKi prior to the risk warning, the proportion of patients who met one or more of these minimization criteria, to better understand the potential impact the new EMA guidelines may have on current and future prescribing of JAKis.

## Method

### Study setting

This analysis was based within the BSR Biologics Register for RA (BSRBR-RA), a UK national prospective cohort study that has been investigating the outcomes in patients starting b/tsDMARDs since 2001 [11]. The BSRBR-RA study was approved by the UK Northwest Multicentre Research Ethics Committee (MREC 00/8/53), and all participants provided written informed consent upon registration in accordance with the Declaration of Helsinki.

### Data collection and definition of variables

Detailed patient data, including date of birth, date of RA diagnosis, gender, smoking status (current smoker, ex-smoker, and never smoked), previous RA treatments, and comorbidities were collected at registration (start of b/tsDMARD therapy). Changes to RA treatment and new adverse events/comorbidities were recorded during follow-up every 6 months for the first 3 years after registration and then annually.

As outlined in the EMA measures to minimize risk, the four criteria for being considered at-risk are: (1) age 65 years or above, (2) increased risk of major cardiovascular problems, (3) current or past smokers, (4) increased risk of cancer. Criterion 1 and 3 are captured in the BSRBR-RA directly. Criterion 2 was operationalized similarly to the ORAL Surveillance inclusion criteria of cardiovascular risk factors and adapted according to data availability, using recorded comorbidities/adverse events (angina, myocardial infarction, stroke, hypertension, diabetes, hyperlipidaemia) or recorded use of lipid-lowering agents, as serum lipid levels are not recorded [4]. Criterion 4 was considered present if the patient had a history of any cancer.

To understand whether any suitable alternative treatment was available, the total number of prior distinct bDMARD classes (TNFis, anti-B cell agents, IL-6is, CTLA4) received prior to starting the JAKi was determined.

### Analysis

The analysis included all patients who started their first-ever JAKi between 13 February 2017 (EMA issue date of first JAKi for RA) and 31 May 2022, the most recent BSRBR-RA data cut-date prior to the publication of the EMA minimization measures on 28 October 2022 [12]. Patients with missing data in any EMA criteria–related variable were excluded. The flow chart of the patient selection is in Supplementary Fig. 1, available at *Rheumatology* online.

Descriptive statistics were used to record patients’ characteristics at the start of their first JAKi. The percentages of patients meeting each individual EMA risk criterion, as well as those meeting multiple criteria, were presented as numbers and percentages overall and stratified by the number of prior bDMARDs classes. All analyses were performed in Stata 14 [13].

## Results

A total of 1341 patients starting their first JAKi were included (78% female, mean age 60 years, mean disease duration 15 years). Baricitinib was the most used (77%), followed by tofacitinib (15%, Table 1). There were 261 patients (19% of total) who commenced a JAKi as their first b/tsDMARDs, while approximately half (49%) of the patients had already had two or more prior classes of bDMARDs, and 27% had already had greater than or equal to three bDMARD classes. In terms of the number of prior bDMARDs, 795 (60%) had received at least two.

**Table 1.** Characteristics and treatment of the 1341 RA patients starting their first JAKi in BSRBR-RA between 13 February 2017 and 31 May 2022

	Missing, *n*	Value
Age in years, mean (s.d.)	0	60 (12)
Female, *n* (%)	0	1048 (78)
Disease duration, years, mean (s.d.)	32	15 (11)
DAS28, mean (s.d.)	492	5.4 (1.2)
Oral glucocorticoids use, *n* (%)	0	352 (26)
First JAKi, *n* (%)		
Baricitinib	0	1039 (77)
Tofacitinib	0	206 (15)
Upadacitinib	0	50 (4)
Filgotinib	0	46 (3)
Number of prior distinct classes of bDMARD, *n* (%)		
0 prior bDMARD classes	0	261 (19)^a^
1 prior bDMARD class	0	419 (31)^a^
2 prior bDMARD classes	0	300 (22)^a^
3 prior bDMARD classes	0	228 (17)^a^
4 prior bDMARD classes	0	133 (10)^a^
Number of prior bDMARDs, *n* (%)		
0 prior	0	255 (19)^a^
1 prior	0	291 (22)^a^
2 prior	0	263 (20)^a^
3 prior	0	240 (18)^a^
4 prior	0	292 (22)^a^
Year of JAKi initiation *n* (%)		
2017	0	59 (4)
2018	0	544 (41)
2019	0	384 (29)
2020	0	151 (11)
2021	0	178 (13)
2022 (until 31 May)	0	25 (2)

a Columns do not add to 100% due to rounding. bDMARDs: biologic DMARDs; BSRBR-RA: BSR Biologics Register for RA; DAS28: 28-joint DAS; JAKi: janus kinase inhibitors.

Eighty percent of all patients (*N* = 1075) met at least one EMA risk criterion, and this percentage remained consistent across different calendar years of JAKi initiation (Table 2). The most common individual criterion met was ever-smokers (54%), followed by cardiovascular risk factors (44%). Around half of the patients (*N* = 637, 47%) met at least two EMA risk criteria. Among those patients who met one or more EMA criteria (*N* = 1075), 196 patients (18%) had received the JAKi as their first b/tsDMARD, whereas 546 (51%) had already received greater than or equal to two bDMARD classes, and 299 (28%) had received greater than or equal to three bDMARD classes. In addition, 61% (654 out of 1075) of patients had received at least two bDMARDs. The proportion of patients who met at least one EMA criterion did not differ in terms of how many prior classes of bDMARDs they had received (75% to 83%, Supplementary Table 1, available at *Rheumatology* online).

**Table 2.** EMA risk criteria among RA patients starting their first JAKi in BSRBR-RA between 13 February 2017 and 31 May 2022

EMA risk criteria	*n* (%)
Patients who had ≥1 EMA risk criterion, (*N* = 1341)	1075 (80)
JAKi initiated in 2017	47 (80)
JAKi initiated in 2018	437 (80)
JAKi initiated in 2019	311 (81)
JAKi initiated in 2020	125 (83)
JAKi initiated in 2021	135 (76)
JAKi initiated in 2022 (until 31^st^ May)	20 (80)
Patients who fulfilled each individual EMA risk criterion, (*N* = 1341)	
(1) Age ≥65	524 (39)
(2) Increased risk of major cardiovascular problems	585 (44)
Hypertension	444 (33)
Hyperlipidaemia	233 (17)
Diabetes	118 (9)
Ischaemic heart disease	75 (6)
Stroke	32 (2)
(3) Smoking status (ever smoker)	727 (54)
Current smokers	235 (18)
Past smokers	492 (37)
(4) Increased risk of cancer	134 (10)
Number of EMA criteria fulfilled, (*N* = 1341)	
0 criterion	266 (20)
1 criterion	438 (33)
2 criteria	399 (30)
3 criteria	218 (16)
All 4 criteria	20 (1)
Number of prior distinct classes of bDMARDs among patients who had ≥1 EMA risk criterion, (*N* = 1075)	
0 prior bDMARD classes	196 (18)^a^
1 prior bDMARD class	333 (31)^a^
2 prior bDMARD classes	247 (23)^a^
3 prior bDMARD classes	188 (17)^a^
4 prior bDMARD classes	111 (10)^a^
Number of prior bDMARDs, among patients who had ≥1 EMA risk criterion, (*N* = 1075)	
0 prior	193 (18)
1 prior	228 (21)
2 prior	214 (20)
3 prior	196 (18)
4 prior	244 (23)

a Columns do not add to 100% due to rounding. bDMARDs: biologic DMARDs, BSRBR-RA: BSR Biologics Register for RA; EMA: European Medicines Agency, JAKi: janus kinase inhibitors.

## Discussion

JAKi prior to publication of the EMA risk warnings, a very high proportion of patients met at least one EMA risk factor. Much of this risk was driven by cardiovascular disease risk factors, indicating the already high level of cardiovascular disease risk–burden in this population.

One of the main challenges of the EMA criteria is how ‘no suitable alternative’ should be interpreted by physicians and patients. Despite four different bDMARD classes being available for RA, several factors impact treatment choice beyond availability of treatment, including comorbidities, contraindications, and adherence preferences (such as tablets over injections). For example, anti-B cell was found to strongly impair vaccine immunogenicity and was perceived as a challenging therapy to manage in light of the COVID-19 risk [14]. In this analysis, 28% of patients with risk factors had already received greater than or equal to three prior bDMARDs, and therefore alternative therapies would be very limited. At the same time, around half the patients who were considered at risk received their JAKi as their first or second b/tsDMARD, suggesting that alternatives could be available. It is unknown whether there were other factors that led to the choice of a JAKi at an earlier stage in these patients, and which may also have meant no other suitable alternatives were available at the time, such as extreme needle phobia or other factors related to adherence. In addition, for those patients who had already received multiple prior bDMARD classes, a large proportion would need discussions with difficult decisions about how to manage their disease control and reduce the potential risk simultaneously.

This analysis was conducted in a large national register-based cohort study with long-term follow-up. While registration into the study is not mandatory, clinicians are recommended by the BSR to register patients receiving these drugs with the appropriate biologics register [15]. The study has wide geographic coverage, and although it does not, nor aim to, capture all b/tsDMARD prescribing nationally, it is considered to be largely representative of the wider UK RA population receiving b/tsDMARDs. Second, detailed risk factors and comorbidities related to the EMA risk criteria were collected, both at registration and during follow-up. There are also some limitations. Some patients starting a JAKi were excluded from this analysis due to missing data on the EMA risk criteria (*N* = 162). Also, the BSRBR-RA does not capture all risk factors related to cardiovascular disease or cancer, such as family history, so some at-risk patients may have been misclassified as not being at risk.

Although all patients in this analysis started their JAKi prior to the publication of the EMA minimization measures to reduce the risk of adverse events in 2022, this was not the only warning issued about JAKis. There were warnings about tofacitinib in particular disseminated even earlier, including several EMA and UK Medicines and Healthcare products Regulatory Agency Direct Healthcare Professional Communications in 2021 [16–19], Oral Surveillance abstracts in 2021 [20], and Oral Surveillance publications in 2022 [4, 6]. As the study can only comment on who did receive a JAKi over this time, it is possible that other patients deemed at higher risk did not start a JAKi at all over this period; however, the prevalence of patients with greater than or equal to one EMA risk criterion in new starters of a JAKi or tofacitinib only (data not shown) did not change over the study period, suggesting that prescribing may not have changed significantly over this period. Most patients in this analysis started baricitinib and not tofacitinib as their first JAKi, and the register does not capture any data on why a particular drug within a class is selected. The nature of the data capture in the BSRBR-RA does not allow one to make any conclusions about the relative prescribing of individual therapies over time.

This large national analysis investigated the potential impact of the measures taken by the EMA to minimize risk of cardiovascular disease and cancer for those using JAKis in the UK. Further analysis of treatment data collected after January 2023 will allow an understanding of the impact of the licensing recommendations and shifts in the practical utilization of JAKis. In summary, a very high proportion of RA patients, four-fifths, commencing a JAKi in the UK prior to the EMA advisory would have fallen into the ‘at-risk’ category for whom prescribing would only be advised if no suitable alternatives were available. Almost a third of those had received three or more prior bDMARDs classes, and for these patients, options for alternative therapies would be very limited. Meanwhile, it is likely that suitable alternatives might have existed for the remaining proportion, and re-evaluation of the suitability of their treatment may be needed. These data highlight the enormous potential impact of the recent licensing updates on treatment with JAKis.

## Supplementary Material

keae279_Supplementary_Data

## Data Availability

Access to the underlying identifiable and potentially re-identifiable pseudonymized electronic health record data is tightly governed by various legislative and regulatory frameworks and restricted by best practice (https://www.rheumatology.org.uk/practice-quality/registers/requesting-registers-data).

## References

[1] McLornan DP , Pope JE, Gotlib J, Harrison CN. Current and future status of JAK inhibitors. Lancet 2021;398:803–16. 34454676 10.1016/S0140-6736(21)00438-4

[2] Winthrop KL , Cohen SB. Oral surveillance and JAK inhibitor safety: the theory of relativity. Nat Rev Rheumatol 2022;18:301–4. 35318462 10.1038/s41584-022-00767-7 PMC8939241

[3] U.S. Food and Drug Administration. Drug approval package. 2012. https://www.accessdata.fda.gov/drugsatfda_docs/nda/2012/203214Orig1s000TOC.cfm (11 June 2012, date last accessed).

[4] Ytterberg SR , Bhatt DL, Mikuls TR et al; ORAL Surveillance Investigators. Cardiovascular and cancer risk with tofacitinib in rheumatoid arthritis. N Engl J Med 2022;386:316–26. 35081280 10.1056/NEJMoa2109927

[5] Kristensen LE , Danese S, Yndestad A et al Identification of two tofacitinib subpopulations with different relative risk versus TNF inhibitors: an analysis of the open label, randomised controlled study ORAL surveillance. Ann Rheum Dis 2023;82:901–10. 36931693 10.1136/ard-2022-223715 PMC10314011

[6] Charles-Schoeman C , Buch MH, Dougados M et al Risk of major adverse cardiovascular events with tofacitinib versus tumour necrosis factor inhibitors in patients with rheumatoid arthritis with or without a history of atherosclerotic cardiovascular disease: a post hoc analysis from ORAL Surveillance. Ann Rheum Dis 2023;82:119–29. 36137735 10.1136/ard-2022-222259 PMC9811099

[7] Salinas CA , Louder A, Polinski J et al Evaluation of VTE, MACE, and serious infections among patients with RA treated with baricitinib compared to TNFi: a multi-database study of patients in routine care using disease registries and claims databases. Rheumatol Ther 2023;10:201–23. 36371760 10.1007/s40744-022-00505-1 PMC9660195

[8] European Medicines Agency’s human medicines committee. EMA confirms measures to minimise risk of serious side effects with Janus kinase inhibitors for chronic inflammatory disorders. 2023. https://www.ema.europa.eu/en/medicines/human/referrals/janus-kinase-inhibitors-jaki (23 January 2023, date last accessed).

[9] National Institute for Health and Care Excellence. Rheumatoid arthritis in adults: management. 2018. https://www.nice.org.uk/guidance/ng100 (12 October 2020, date last accessed). 30102507

[10] Zhao SS, , Kearsley-Fleet L, , Bosworth A, , Watson K, , Hyrich KL. Effectiveness of sequential biologic and targeted disease modifying anti-rheumatic drugs for rheumatoid arthritis. Rheumatology 2022;61:4678–86. 35357421 10.1093/rheumatology/keac190 PMC9707051

[11] Hyrich K L, , Watson K D, , Isenberg D A, , Symmons DPM. The British Society for Rheumatology biologics register: 6 years on. Rheumatology 2008;47:1441–3. 18596052 10.1093/rheumatology/ken242

[12] European Medicines Agency’s Pharmacovigilance Risk Assessment Committee. EMA recommends measures to minimise risk of serious side effects with Janus kinase inhibitors for chronic inflammatory disorders. 2022. https://www.ema.europa.eu/en/news/ema-recommends-measures-minimise-risk-serious-side-effects-janus-kinase-inhibitors-chronic (28 October 2022, date last accessed).

[13] StataCorp. Stata Statistical Software: Release 14. College Station, TX: StataCorp LLC 2015. https://www.stata.com/support/faqs/resources/history-of-stata/ (3 June 2024, date last accessed).

[14] Garcillán B, , Salavert M, , Regueiro JR, , Díaz-Castroverde S. Response to vaccines in patients with immune-mediated inflammatory diseases: a narrative review. Vaccines 2022;10:297. 35214755 10.3390/vaccines10020297 PMC8877652

[15] Holroyd CR , Seth R, Bukhari M et al The British Society for Rheumatology biologic DMARD safety guidelines in inflammatory arthritis. Rheumatology 2019;58:e3–e42. 30137552 10.1093/rheumatology/key208

[16] European Medicines Agency. Xeljanz (tofacitinib): increased risk of major adverse cardiovascular events and malignancies with use of tofacitinib relative to TNF-alpha inhibitors. 2021. https://www.ema.europa.eu/en/documents/dhpc/direct-healthcare-professional-communication-dhpc-xeljanz-tofacitinib-increased-risk-major-adverse-cardiovascular-events-and-malignancies-use-tofacitinib-relative-tnf-alpha-inhibitors_en.pdf (6 July 2021, date last accessed).

[17] European Medicines Agency. Xeljanz—direct healthcare professional communication (DHPC). 2021. https://www.ema.europa.eu/en/medicines/dhpc/xeljanz (24 March 2021, date last accessed).

[18] Medicines and Healthcare products Regulatory Agency. Tofacitinib (Xeljanz): new measures to minimise risk of major adverse cardiovascular events and malignancies. 2021. https://www.gov.uk/drug-safety-update/tofacitinib-xeljanzv-new-measures-to-minimise-risk-of-major-adverse-cardiovascular-events-and-malignancies (6 October 2021, date last accessed).

[19] Medicines and Healthcare Products Regulatory Agency. Xeljanz (tofacitinib): Increased risk of major adverse cardiovascular events and malignancies with use of tofacitinib relative to TNF-alpha inhibitors. 2021 https://assets.publishing.service.gov.uk/media/611669388fa8f53dc688bcbb/Xeljanz_DHPC_230721.pdf (23 July 2021, date last accessed).

[20] Ytterberg S , Bhatt D, Mikuls T et al Safety and efficacy of tofacitinib vs TNF inhibitors in RA patients aged 50 years or older with one or more cardiovascular risks: results from a phase 3b/4 randomized safety trial. Arthritis Rheumatol 2021. https://acrabstracts.org/abstract/safety-and-efficacy-of-tofacitinib-vs-tnf-inhibitors-in-ra-patients-aged-50-years-or-older-with-one-or-more-cardiovascular-risks-results-from-a-phase-3b-4-randomized-safety-trial/ (3 June 2024, date last accessed).

