# Supplementary material for: Potential impact of European Medicines Agency measures to minimize risk of serious side effects on JAKi prescribing and utilization in the UK
Source: Rheumatology (Oxford). 2024 May 15;64(3):1453–8. doi: 10.1093/rheumatology/keae279 (PMC11879295; doi:10.1093/rheumatology/keae279)
Supplement: keae279_Supplementary_Data [file keae279_supplementary_data.docx]

# Supplementary Materials

Receiving first JAKi during their participation in BSRBR-RA [N=1 524]

RA patients recruited to BSRBR-RA [N=27 241]

Start b/tsDMARDs before and on 31st May 2022 [N=22 271]

Diagnosis of RA was above 16 years old [N=21 923]

Receiving JAKi [N=1 550]

Receiving first JAKi after 13-February-2017

[N=1 503]

With complete data in all EMA criteria related variables

[N=1 341]

### Supplementary Figure S1: Patient inclusion

N for patient number

BSRBR-RA: British Society for Rheumatology Biologics Register for RA, b/tsDMARDs: biological or targeted synthetic disease-modifying anti-rheumatic drugs, EMA: European Medicines Agency, JAKi: Janus kinases inhibitors; RA: rheumatoid arthritis

Supplementary Table S1 EMA risk criteria among RA patients starting their first JAKi in BSRBR-RA between 13th February 2017 and 31st May 2022, stratified by the number of prior distinct bDMARDs classes

| EMA risk criteria | Patients at risk by the number of prior distinct classes of bDMARDs | | | | |  |
| --- | --- | --- | --- | --- | --- | --- |
|  | 0 prior | 1 prior | 2 prior | 3 prior | 4 prior |  |
| Total number of patients in each stratum, N | 261 | 419 | 300 | 228 | 133 |  |
| Total number of patients who meet ≥1 EMA risk criterion, n% | 196 (75) | 333 (80) | 247 (82) | 188 (83) | 111 (83) |  |
| (1) Age ≥65 | 92 (35) | 167 (40) | 134 (45) | 87 (38) | 44 (33) |  |
| (2) Increased risk of major cardiovascular problems | 99 (38) | 177 (42) | 137 (46) | 100 (44) | 72 (54) |  |
| Hypertension, n (%) | 64 (25) | 148 (35) | 107 (36) | 78 (34) | 47 (35) |  |
| Hyperlipidaemia, n (%) | 49 (19) | 61 (15) | 48 (16) | 42 (18) | 33 (25) |  |
| Diabetes, n (%) | 21 (8) | 38 (9) | 31 (10) | 16 (7) | 12 (9) |  |
| Ischemic heart disease, n (%) | 9 (3) | 20 (5) | 20 (7) | 16 (7) | 10 (8) |  |
| Stroke, n (%) | 5 (2) | 9 (2) | 6 (2) | 7 (3) | 5 (4) |  |
| (3) Smoking status | 140 (54) | 225 (54) | 154 (51) | 129 (57) | 79 (59) |  |
| Current smokers | 49 (19) | 59 (14) | 46 (15) | 48 (21) | 33 (25) |  |
| Past smokers | 91 (35) | 166 (40) | 108 (36) | 81 (36) | 46 (35) |  |
| (4) Increased risk of cancer | 25 (10) | 33 (8) | 36 (12) | 21 (9) | 19 (14) |  |
| bDMARDs: biological disease-modifying anti-rheumatic drugs, BSRBR-RA: British Society for Rheumatology Biologics Register for Rheumatoid Arthritis; EMA: European Medicines Agency, JAKi: janus kinase inhibitors, RA: rheumatoid arthritis. | | | | | | |
